# Probing Molecular Insights into Zika Virus–Host Interactions

**DOI:** 10.3390/v10050233

**Published:** 2018-05-02

**Authors:** Ina Lee, Sandra Bos, Ge Li, Shusheng Wang, Gilles Gadea, Philippe Desprès, Richard Y. Zhao

**Affiliations:** 1 Department of Pathology, University of Maryland School of Medicine, Baltimore, MD 21201, USA; ilee1@umm.edu (I.L.); GLi@som.umaryland.edu (G.L.); shushengwang018@gmail.com (S.W.); 2 Université de la Réunion, INSERM U1187, CNRS UMR 9192, IRD UMR 249, Unité Mixte Processus Infectieux en Milieu Insulaire Tropical, Plateforme Technologique CYROI, 94791 Sainte Clotilde, La Réunion, France; sandrabos.lab@gmail.com (S.B.); gilles.gadea@inserm.fr (G.G.); philippe.despres@univ-reunion.fr (P.D.); 3 Department of Microbiology and Immunology, University of Maryland School of Medicine, Baltimore, MD 21201, USA; 4 Institute of Global Health, University of Maryland School of Medicine, Baltimore, MD 21201, USA; 5 Institute of Human Virology, University of Maryland School of Medicine, Baltimore, MD 21201, USA

**Keywords:** zika virus, ZIKV–host interactions, viral pathogenesis, cell surface receptors, antiviral responses, viral counteraction, cytopathic effects, microcephaly, ZIKV-associated neurologic disorders

## Abstract

The recent Zika virus (ZIKV) outbreak in the Americas surprised all of us because of its rapid spread and association with neurologic disorders including fetal microcephaly, brain and ocular anomalies, and Guillain–Barré syndrome. In response to this global health crisis, unprecedented and world-wide efforts are taking place to study the ZIKV-related human diseases. Much has been learned about this virus in the areas of epidemiology, genetic diversity, protein structures, and clinical manifestations, such as consequences of ZIKV infection on fetal brain development. However, progress on understanding the molecular mechanism underlying ZIKV-associated neurologic disorders remains elusive. To date, we still lack a good understanding of; (1) what virologic factors are involved in the ZIKV-associated human diseases; (2) which ZIKV protein(s) contributes to the enhanced viral pathogenicity; and (3) how do the newly adapted and pandemic ZIKV strains alter their interactions with the host cells leading to neurologic defects? The goal of this review is to explore the molecular insights into the ZIKV–host interactions with an emphasis on host cell receptor usage for viral entry, cell innate immunity to ZIKV, and the ability of ZIKV to subvert antiviral responses and to cause cytopathic effects. We hope this literature review will inspire additional molecular studies focusing on ZIKV–host Interactions.

## 1. Introduction

### 1.1. The Zika Virus (ZIKV): An Emerging Public Health Threat

The 2015 Zika virus (ZIKV) epidemic in the Americas surprised the world because of its rapid global spread and the findings that it associates with various neurologic disorders including microcephaly in newborns and Guillain–Barré syndrome (GBS) in adults. ZIKV was thought to be a mild virus that had little or no threat to humans. Through studies of this new ZIKV pandemic, we have now learned that ZIKV is a rather severe human pathogen that can cause significant neuropathology such as fetal microcephaly, GBS, and various other congenital neurologic and ocular disorders [1,2,3,4,5]. So, it begs the question of what has transformed a benign ZIKV over the past seventy years to generate the contemporary pathogenic ZIKV.

The goal of this article is to review the current literature on ZIKV–host interactions with a focus on molecular aspects. We herein summarize insights on host cell receptor usage for viral entry, cell innate immunity to ZIKV, and the ability of ZIKV to subvert antiviral responses and to cause cytopathic effects. The molecular mechanisms underlying these ZIKV–host interactions, and their potential impacts on ZIKV-induced fetal microcephaly or other neurologic disorders are discussed.

### 1.2. The Organization of Zika Virus

**Figure 1:**
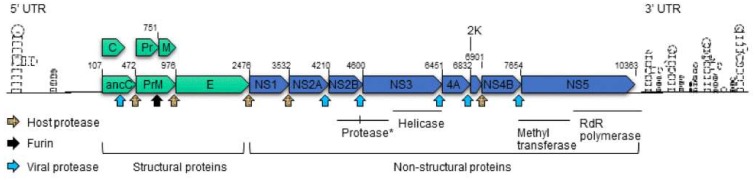
Schematic structure of the Zika virus genome. Each of the viral proteins is drawn based on the relative orientation in the RNA genome. The ZIKV viral protease, host protease and Furin protease are represented by different arrows, as shown. Each arrow points to the specific protease cleavage site. The numbers shown above each protein product indicate the start/end position. Abbreviations: anaC, anchored capsid protein C; C, capsid protein C; prM, precursor membrane protein; M, membrane protein; Pr, protein pr; E, envelope protein; NS, nonstructural protein; *, protease consists of N-terminal of NS3 and C-terminal NS2B as described in the text. C-terminal of NS3 encodes helicase; 2K, signal peptide 2K; NS5 encodes methyltransferase at its N-terminal end and RNA-dependent RNA (RdR) polymerase at its C-terminal end. UTR, untranslated region. The structures of 5′ UTR and 3′ UTR are based on [21]. The information of ZIKV protein products is based on [9].

ZIKV belongs the *flavivirus* genus of the *Flaviviridae* family which includes a number of medically important arboviruses such as Dengue Virus (DENV), Japanese Encephalitis Virus (JEV), and West Nile Virus (WNV). Structurally, ZIKV is similar to other flaviviruses. The nucleocapsid is approximately 25–30 nm in diameter, surrounded by a host membrane-derived lipid bilayer that contains envelope (E) and membrane (M) proteins. The virus particle is approximately 40–65 nm in diameter, with surface projections that measure roughly 5–10 nm [6], leading an overall average size of 45–75 nm. The surface proteins are arranged in an icosahedral-like symmetry [7]. Like its flaviviral siblings, ZIKV contains a positive sense single-stranded RNA [ssRNA(+)] viral genome of approximately 10.7 kilobases (kb) (Figure 1). The genomic RNA is flanked by two terminal non-coding regions (NCR), i.e., the 5′ NCR (107 nt) and the 3′ NCR (428 nt) [8]. The ZIKV genome includes a single large open reading frame encoding a polyprotein of about 3300 amino acids, which is processed co- and post-translationally by viral and host proteases (PRs) to produce a total of fourteen immature proteins, mature proteins, and small peptides [9]. A total of ten mature viral proteins, i.e., three structural proteins (C, M, and E) and seven nonstructural (NS1, NS2A, NS2B, NS3, NS4A, NS4B, and NS5) proteins are produced after viral processing [6,9,10]. The 2K signal peptide, which is situated between NS4A and NS4B, plays a regulatory role in viral RNA synthesis and viral morphogenesis in other flaviviruses [11,12]. Among the structural proteins, the mature capsid (C) protein is produced by cleavage of the anchor-C (anaC) protein by a viral PR (anaC→C), which in turn triggers the cleavage of the precursor membrane (prM) protein by the host protease Furin. As a result, a mature membrane (M) protein and a Pr protein are produced (PrM→M + Pr) [11,13]. In the case of DENV, noninfectious and immature viral particles contain prM that forms a heterodimer with the E protein [14]. The transition of prM to M by Furin cleavage results in mature and infectious particles [15,16]. The E protein, composing the majority of the virion surface, is involved in binding to the host cell surface and triggering subsequent membrane fusion and endocytosis [8]. Post-translational processing of the non-structural protein region produces four viral enzymes, i.e., PR, helicase, methyl-transferase, and RNA-dependent RNA polymerase (RdRP). A fully active ZIKV PR consists of two protein components, namely the N-terminal domain of NS3 (NS3pro) and a membrane-associated NS2B cofactor [17,18]. The NS3pro is responsible for proteolytic processing of the viral polyprotein, whereas the NS2B cofactor is required for enhancing enzymatic activity and substrate specificity. The C-terminal domain of NS3 protein produces ZIKV helicase, which plays a critical role in NTP-dependent RNA unwinding and translocation during viral replication [19]. The methyl-transferase and RdRP are generated from the N-terminal and C-terminal of NS5, respectively. NS1, NS3, and NS5 are large proteins that are highly-conserved [6]. NS2A, NS2B, NS4A, and NS4B proteins are smaller, hydrophobic proteins [6]. The 3′ NCR forms a loop structure that may play a role in translation, RNA packaging, cyclization, genome stabilization and recognition [8]. The 5′ NCR allows translation via a methylated nucleotide cap or a genome-linked protein [7]. In addition, ZIKV produces abundant non-coding subgenomic flavivirus RNA (sfRNA) from the 3′UTR in infected cells, which may play a role in the viral life cycle and viral subversion of innate immunity [20].

### 1.3. The Infectious Cycle of ZIKV and Human Transmission 

The ZIKV infectious cycle starts with the virus binding to host cell surface receptors and attachment factors via the E protein, leading to clathrin-dependent endocytosis. Internalized virus particles fuse with the endosomal membrane in a pH-dependent manner, releasing the genomic RNA into the cytoplasm of the host cell. The positive-sense viral genomic ssRNA is translated into a polyprotein that is subsequently cleaved to form structural and non-structural proteins. Viral replication takes place in intracellular membrane-associated compartments located on the surface of the endoplasmic reticulum (ER), resulting in a dsRNA genome synthesized from the genomic ssRNA(+) by viral RdRP. The dsRNA genome is subsequently transcribed and replicated, resulting in additional viral mRNAs/ssRNA(+) genomes. Immature virus particles are assembled within ER. They are then transported through the trans-Golgi network (TGN) where the fully mature infectious virus particles are formed as soon as prM is processed to M by a Furin-like protease. The new virus particles are released into the extracellular environment where they move on to a new infectious life cycle [7].

ZIKV is an arbovirus that is primarily transmitted to mammalian hosts by mosquito vectors from the *Aedes (Ae.)* genus including *Ae. africanus*, *Ae. aegypti*, *Ae. vitattus*, *Ae. furcifer*, *Ae. apicoargenteus*, and *Ae. luteocephalus* [22,23]. The incubation time for ZIKV in mosquito vectors is approximately 10 days [22]. Blood contact via blood transfusion or sexual contact is another route of ZIKV infection [24,25,26]. Consistent with this notion, human testis has been found to be a reservoir for ZIKV [22,27,28,29]. In addition, ZIKV can also be transmitted vertically from mother to child via placenta–fetal transmission [30,31]. This has become a main route for the development of fetal microcephaly [32,33].

### 1.4. A Brief History of ZIKV 

The first ZIKV strain was isolated in 1947 from caged monkeys in the Zika forest of Uganda, Africa. A ZIKV strain MR766 (ZIKV_MR766_) from that isolation was established and has been used since for research purpose [34]. Therefore, the ZIKV_MR766_ is often referred to as the historical or ancestral ZIKV strain. Initial characterization of ZIKV_MR766_ showed that it is highly neurotropic in mice, and no virus has been recovered from tissues other than the brains of infected mice [35]. That report further showed that mice at all ages were susceptible to ZIKV_MR766_ by intracerebral inoculations. In contrast, cotton-rats, Guinea pigs, and rabbits showed no sign of ZIKV_MR766_ infection by using the same intracerebral inoculations. Monkeys showed either mild fever (pyrexia) or no signs of infection. Interestingly, mice younger than two weeks were highly susceptible to intraperitoneal inoculation, whereas mice older than two weeks can rarely be infected by the same route of intraperitoneal injection [35], suggesting established blood-brain barriers in the older mice may prevent ZIKV from accessing the brain.

In a different study, the effect of ZIKV infection on the central nervous system (CNS) of mice was examined by using intracerebral inoculation [36]. Histologic H & E staining showed that ZIKV infects the Ammon’s horn (hippocampus proper) area of seven-day-old mouse brain. Detailed examination suggested that ZIKV infected pyriform cells of the Ammon’s horn and induced hyperchromatic debris in those cells, suggesting possible DNA or chromosomal aberration. In addition, ZIKV induced gross enlargement (hypertrophy) of astroglial cells of the Ammon’s horn, but had little effect on microglial cells of the same area [36]. Ultra-structural studies by electron microscopy (EM) further revealed that ZIKV replicates exclusively in the ER compartment of astroglial cells and neurons, an indication of membrane-associated viral replication [36].

Those early findings in the mouse model suggest that (1) ZIKV is a neurotropic virus with preference to embryonic brains [34,35], (2) ZIKV specifically infect astroglial cells and pyriform cells in the Ammon’s horn, and (3) ZIKV primarily replicates in the ER network [36]. At the cellular level, ZIKV appeared to induce gross cell enlargement, chromosomal or DNA aberrations, and mitochondrial dysfunction [36]. Although early data showed that ZIKV was pathogenic to mice, there was no indication that ZIKV was pathogenic to humans [35]. Therefore, some types of virological change are likely to have taken placed during the viral evolution in the past seventy years, leading to pathogenic ZIKV infection of humans.

The first ZIKV infection in human was documented in 1952 [34], and the virus was subsequently isolated from human hosts in Nigeria in 1968 [22]. Since then, multiple studies have confirmed the presence of ZIKV antibodies in human sera from a number of countries in Africa and Asia [22]. However, no severe diseases were clearly linked to those infections. In the recorded history, ZIKV infection appears to have migrated eastward from Africa. A number of outbreaks have taken place over the past seventy years including several minor outbreaks in 1977–1978 in Pakistan, Malaysia, and Indonesia. Two major outbreaks were documented in Yap Island of Micronesia in 2007, and in French Polynesia, New Caledonia, the Cook Islands, and Easter Island in 2013 and 2014 [26,37]. The affected individuals in those outbreaks were in the order of hundreds to thousands. However, in the most recent outbreak, ZIKV infection had been reported in eighty-five countries, territories, or subnational areas with an estimate of over 1.5 million affected individuals according to the World Health Organization (WHO). Brazil was the most affected country, with an estimated 440,000 to 1.3 million cases reported through December of 2015.

Although human ZIKV infection is mostly self-limiting, manifestations of neurological disorders such as GBS became increasingly apparent during the recent outbreaks in French Polynesia and Brazil [32,38]. The number of microcephaly in newborns also increased dramatically, which for the first time indicated a possible link between ZIKV infections and fetal malformations [32,39,40]. More than 4700 suspected cases of microcephaly were reported from mid-2015 through to January 2016 [41], spurring an unprecedented and world-wide effort to unravel this mystery. By March of 2016, the causal relationship between microcephaly in newborn and ZIKV infection was first established [32,39,40]. By April of 2016, a total of 3530 newborns with confirmed microcephaly were reported. In the same year, WHO declared an international public health emergency. In-depth research now shows that ZIKV infection is also associated with a number of other congenital and ocular diseases [1,2].

### 1.5. What Has Been Learned from the Recent ZIKV Break?

We have learned a great deal about the ZIKV and its etiology through the above described studies. The knowledge we have gained is that fetal microcephaly and other congenital malformations can indeed be caused by ZIKV infection [32,42,43]. Furthermore, those circulating and pathogenic ZIKV strains are most likely derived from the Asian lineage [44,45]. The Asian lineage is likely evolved from the African lineage through viral gene mutations by adaptation of higher cytopathicity that led to enhanced viral pathogenicity. Particular efforts have been put to investigate whether the emergence of new ZIKV epidemic strains was associated with accumulation of specific mutations that would be the leading cause of increased pathogenic effects [44,45,46]. Also, investigations have been conducted to determine whether pathogenic strains of ZIKV could preferentially infect certain human tissues or cells, especially neural progenitor cells (NPCs) in the brain, or they have acquired greater virulence through accumulated effects of ZIKV gene mutations [47,48,49]. The antibody-dependent enhancement (ADE) may also contribute to the acquired virulence [50]. This scenario could occur if individuals have previously been exposed to other flaviviruses and have acquired antibodies that partially cross-react with ZIKV. Instead of neutralizing ZIKV, these antibodies could paradoxically argument ZIKV infectivity [50]. As a matter of fact, the opposite scenario has been observed in which pre-exposure of ZIKV was associated with enhanced DENV-2 infection in vitro [51] and in monkeys [52]. Therefore, enhanced ZIKV infection as the result of prior exposure of other flaviviral infection could certainly be feasible [53,54,55,56]. However, ADE is less likely to be the predominant mode of enhanced ZIKV pathogenicity in the recent break since ZIKV is known to cause fetal microcephaly in the absence of antibody response to other flaviviruses. We should also be mindful that despite these theories, we cannot exclude another possibility that ZIKV-induced microcephaly may not be the result of ZIKV evolution, but rather a reflection of the advanced technology in disease monitoring and diagnosis. In other words, microcephaly is intrinsic to all ZIKV strains but its evasion from public awareness could be due to the lack of sensitive detection methods in the past. This possibility may not totally be far-fetched, insofar that the very first ZIKV isolate, ZIKV_MR766_, also induced microcephaly in animal and human brain-specific organoid models [40,43,57]. In fact, both African and Asian ZIKV strains (MR766, FSS13025, PF/2013/KD507, SZ01, and various epidemic Brazilian strains, e.g., ZIKV-BR/2015), have been shown to induce microcephaly-like phenotypes in animal and human brain-specific organoid models (Table 1) [40,43,57,58,59]. Nevertheless, virological activities of the ancestral ZIKV_MR766_ did appear to be different from Asian lineage in embryonic mouse brains [60]. Therefore, as it says that the devil is in the detail. It is very likely that the neurological defects caused by the epidemic Brazilian ZIKV in humans were attributed by subtle but important virological changes. Those newly adapted virological changes could include preferable infection to certain brain and neural cells such as hNPCs, persistent viral replication in host cells, and enduring neuropathic damages that lead to those observed ZIKV-associated neurological disorders. Further and detailed dissections of those virological traits certainly are warranted.

In short, even though we have learned a great deal about the ZIKV etiology, much still remains unknown. Some of the critical questions include; (1) what type of virological changes have taken place to result in increased viral pathogenicity, (2) which ZIKV protein(s) is responsible for the enhanced viral pathogenicity, and (3) how do the newly adapted ZIKV strains alter their interactions with host cells that lead to those neurologic defects? In particular, the specific mechanisms underlying the molecular actions of ZIKV-mediated neurologic disorders such as microcephaly and other neurologic disorders need to be thoroughly investigated.

Viral pathogenicity is normally referred to the state of a virus and its ability to cause disease. The attributes of viral pathogenicity are often constituted by the target of organ, tissue, and cells (cell tropism), the level and persistence of viral replication in host cells, and the ability of the virus to cause damage to host cells that is referred to as cytopathic effects (CPEs). Both historical and contemporary ZIKV strains have the capacity to replicate in brain-specific neuronal cells [34]. However, so far, only the epidemic strains were associated to congenital fetal microcephaly and other neurologic disorders, highlighting that viral factors other than the cell tropism are more likely contributing to the increased viral pathogenicity. Furthermore, multifactorial viral functions might have contributed to those ZIKV-associated diseases. Conceivably, it could be the changing balance in ZIKV–host interactions that leads to favorable and persistent ZIKV viral replication in host cells such as hNPCs, increased and lasting CPEs that ultimately contribute to those observed fetal development and neurologic disorders. In the following sections, we will discuss the molecular aspects of ZIKV–host interactions, which include (1) host target cells and cell surface receptors for viral entry, (2) host cellular and immune responses to ZIKV replications, (3) counteracting effects of ZIKV to host antiviral responses, and (4) ZIKV-induced cytopathic effects (restricted cell growth, cell cycle dysregulation, and cell death/apoptosis) that are all known contributing factors to fetal brain development and neurologic impairments [42,57,61].

## 2. Cellular Targets and Viral Entry

### 2.1. Cellular Targets

ZIKV primarily infects NPCs in embryonic brains [42,61,63]. In the adult brain, it also infects astroglial and microglial cells, and to lesser extent, neurons [36,42]. In addition, ZIKV infects other tissues such as skin (including dermal fibroblasts and epidermal keratinocytes), testis, and placenta (Table 2). As an arbovirus, ZIKV transmission is predominately through skin by mosquitoes such as *Ae. aegypti* and *Ae. africanus* [22,23]. Consistent with this route of transmission, immature and mature dendritic cells are susceptible to ZIKV infection [64,65,66]. ZIKV can also be transmitted through sexual contacts [24,25,26]. Infected Sertoli cells in human testis are known ZIKV reservoirs [27,28,29]. Several placenta-specific cells have been shown to be prone to ZIKV infection including Hofbauer cells, trophoblasts, and placental endothelial cells, supporting an important role of the placenta in transmitting ZIKV via blood to fetal brains [33,67,68]. In line with the idea that crossing the blood-brain barrier might be required to transmit the virus to the brain compartment [35], ZIKV persistently infects primary human brain microvascular endothelial cells (hBMECs) or established cell lines [69]. Interestingly, a hepatoma cell line Huh-7 appears to be highly permissive to ZIKV infection. However, liver has not yet been documented to be the target organ of ZIKV, even though DENV is well-known to infect liver [70,71].

### 2.2. The Cellular Receptors for ZIKV Entry

Flaviviruses enter host cells by clathrin-dependent endocytosis, which is initiated when viral particles interact with cell surface receptors. The cell surface receptors bind to the infectious viral particles and direct them to the endocytic pathway. Several cell surface receptors facilitate ZIKV viral entry (Table 2), which include the tyrosine-protein kinase receptor AXL, Tyro3, DC-SIGN, and TIM-1 [64,65]. AXL and Tyro3 are part of the TAM receptor tyrosine kinase family that normally binds to Gas6 and Pros1 ligands. These receptors are known to regulate an array of cellular activities including cell adhesion, migration, proliferation, and survival, as well as the release of inflammatory cytokines, which play pivotal roles in innate immunity [72]. DC-SIGN is an innate immune receptor present on the surface of both macrophages and dendritic cells (DCs). It recognizes a broad range of pathogen-derived ligands and mediates antigen uptake and signaling [73]. The TIM-1 receptor, also known as HAVcr-1 (Hepatitis A virus cellular receptor 1), plays an important role in host response to viral infection.

Even though all of the aforementioned cell surface receptors participate in ZIKV viral entry, they are not unique to ZIKV infection. For example, AXL, Tyro3, and DC-SIGN are used by Lassa virus [74]. The TIM-1 receptor mediates infections of the deadly Ebola virus [75]. In fact, both TAM and TIM families of phosphatidylserine receptors also mediate viral entry of other flaviviruses such as DENV [76] and WNV [77]. For instance, in the case of DENV, TIM receptors facilitate viral entry by directly interacting with virus-associated phosphatidylserine, whereas TAM-mediated infection relies on indirect viral recognition, in which the TAM ligand Gas6 acts as a bridging molecule by binding to phosphatidylserine within the viral particle [76]. Reviews of this topic can be found in [78,79].

Involvement of AXL, Tyro3, DC-SIGN, and, to a lesser extent, TIM-1 was initially described by Hamel et al. when they studied ZIKV entry in skin cells [64]. AXL was subsequently shown to be a prime target receptor for ZIKV viral entry in a variety of cell types including human endothelial cells (hECs) [61], neural stem cells [80], microglia and astrocytes [81], and oligodendrocyte precursor cells [82]. Examination of the AXL expression levels of diverse cell types suggests that AXL is highly expressed on the surface of human radial glial cells, astrocytes, hECs, oligodendrocyte precursor cells, and microglia in the developing human cortex as well as in progenitor cells of the developing retina [80,82]. Other ZIKV permissive and non-neuronal human cell types, which are known to express AXL, Tyro3, and/or TIM1 and likely to mediate viral entry, include placental cells, explants-cytotrophoblasts, endothelial cells, fibroblasts, and Hofbauer cells in chorionic villi as well as amniotic epithelial cells and trophoblast progenitors in amniochorionic membranes [83].

The susceptibility of human ECs to ZIKV positively correlates with the cell surface levels of AXL [61]. Gain- and loss-of-function tests revealed that AXL is required for ZIKV entry at a post-binding step, and small-molecule inhibitors of the AXL kinase significantly reduced ZIKV infection of hECs [61]. In human microglia and astrocytes of the developing brain, like DENV, AXL-mediated ZIKV entry requires the AXL ligand Gas6 to serve as a bridge linking ZIKV particles to glial cells [81]. Following binding, ZIKV is internalized through clathrin-mediated endocytosis and is transported to Rab5+ endosomes to establish productive infection. Downregulation of AXL by an AXL inhibitor R428 or an AXL decoy receptor MYD1 significantly reduced but did not abolish the ZIKV infection, suggesting the AXL receptor might be the primary but not the only receptor that is required for ZIKV infection [81]. Genetic knockdown of AXL in a glial cell line nearly abolished ZIKV infection [82]. It should be mentioned that elimination of any known entry receptor does not result in complete protection from viral infection, as flaviviruses use many different receptors, there is always redundancy and alternatives.

Interestingly, genetic ablation of the AXL receptor by CRISPR/CAS9 did not protect hNPCs and cerebral organoids from ZIKV Infection [84]. In particular, genetic ablation of AXL has no effect on ZIKV entry or ZIKV-mediated cell death in human induced pluripotent stem cell (iPSC)-derived NPCs or cerebral organoids. It is not yet clear what contributes to the observed discrepancy between this and other studies. One possibility is that ZIKV may use different cell surface receptors on iPSC-derived NPCs [84]. For example, TIM-1 plays a more prominent role than AXL in placental cells [83]. Duramycin, a peptide that binds phosphatidylethanolamine in enveloped viral particles and precludes TIM1 binding, reduced ZIKV infection in placental cells and explants. In a mouse study, comparison of homozygous or heterozygous AXL knock-out showed no significant differences in ZIKV viral replication and clinical manifestation, suggesting AXL is dispensable for ZIKV infection in those mice [85].

## 3. Cellular and Immune Responses to ZIKV Infection

**Figure 2:**
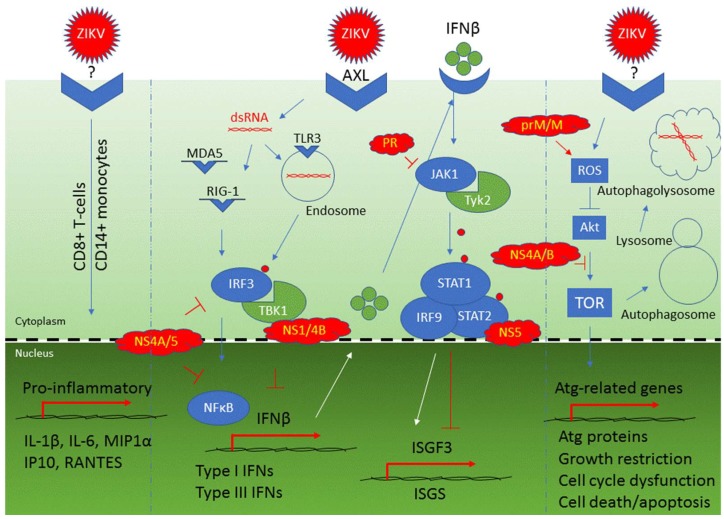
This figure illustrates Zika virus interactions with host cells. The Zika virus or proteins are colored in red. Cellular receptors or proteins that are affected by ZIKV are shown in blue. Cellular proteins shown in green are regulatory proteins such as kinases. Three Zika viruses are used here to show ZIKV-induced T-cell responses (left), ZIKV-mediated type I and type III IFNs productions (middle) and ZIKV-triggered autophagy (right). → indicates a positive interaction. ┤ denotes inhibitory action. Small red dots are used to indicate phosphorylation.

Inflammation is one of the first line responses of the cellular immune system to viral infection, which is typically ignited by releasing cytokines including chemokines (Table 3). ZIKV triggers various host cell pro-inflammatory responses (Figure 2) [64,65,101,103]. For example, ZIKV stimulates CD8+ T cell-mediated polyfunctional immune responses to induce NF-κB-mediated production of cytokines such as IL-1β, IL-6, MIP1α, as well as chemokines including IP10 and RANTES [87,103] (Figure 2, left). These ZIKV-induced T cell immune responses are antiviral because when CD8+ isolates from previously ZIKV infected mice are introduced to naive mice prior to ZIKV infection, viral clearance is enhanced. Conversely, depletion of CD8+ T cells from infected animals compromises viral clearance [65]. ZIKV structural proteins (C, prM, and E) are the major targets of CD8+ T cell and CD4+ T cell responses [104].

ZIKV also elicits humoral immune responses by producing protective and neutralizing antibodies in humans [34,45]. However, this antibody-mediated protection effect against ZIKV could be jeopardized in individuals who have previously been exposed to other flaviviruses such as DENV, which is the closest sibling of ZIKV. Pre-existing neutralizing antibodies against DENV presented in those individuals could, instead of neutralizing ZIKV, actually augment ZIKV infection and lead to more severe diseases [50]. This ADE effect of prior flaviviral infections on ZIKV pathogenicity has been thoroughly reviewed elsewhere [105,106].

Aside from ZIKV-mediated inflammatory and humoral responses, ZIKV also triggers a series of host cell innate immune responses, which are crucial for the recognition of viral invasion, activation of antiviral responses, and determination of the fate of viral infected cells (Figure 2, middle). Generally, primed by the pathogen-associated molecular pattern (PAMP) of different viruses, host cells recognize the invading virus by activating different types of pattern recognition receptors (PRRs), which could be cell surface receptors or endosomal receptors. For example, ZIKV is recognized by an endosomal toll-like receptor 3 (TLR3), which is a PRR that specifically recognizes dsRNA virus [57,64,65]. TLR3 belongs to a class of endosomal receptors that can be found in first line of defense cells such as macrophages or Langerhans cells. TLR3 activation plays a key role in host cell innate immune responses to viral infection. Consistent with the innate immune responses to dsRNA virus, ZIKV-induced TLR3 activation promotes phosphorylation of interferon regulatory factor 3 (IRF3) by TBK1 kinase, leading to induction of type 1 interferon (IFN) signaling pathways [65,107]. This initiates a cascade that further activates cytoplasmic RIG-I-like receptors (RLRs) responses, subsequently inducing transcription of RIG-I, MDA5, and several type I and III IFN-stimulated genes including OAS2, ISG15, and MX1 [64]. Activation of the type I IFN signaling pathway results in production and secretion of IFN-β. Secreted IFN-β binding to IFN-β receptor activates JAK1 and Tyk2 kinases that in turn phosphorylate STAT1 and STAT2 (Figure 2, middle). Upon ZIKV infection, association of the phosphorylated STAT1/STAT2 heterodimer with IRF9 promotes ISRE3-mediated transcription of antiviral interferon stimulated genes (ISGS) [65]. One of the ISGS proteins, viperin (virus-inhibitory protein, endoplasmic reticulum-associated, IFN-inducible), shows strong antiviral activity against ZIKV. Specifically, it restricts ZIKV viral replication by targeting the NS3 protein for proteasomal degradation [108]. Therefore, the production of TLR3- and RIG-1/MDA5-mediated type I IFN production and subsequent activation of the JAK/STAT innate immune pathway confer increased resistance to ZIKV infection [109].

ZIKV is a membrane-associated virus that utilizes host ER for its replication and reproduction along the cellular secretory pathway. Through those cellular membrane interactions, ZIKV can trigger autophagy in a cell-dependent manner (Figure 2, right). This cellular process is normally involved in removal of aggregated or erroneously folded proteins through lysosomal degradation. Activation of cellular autophagy is a hallmark of flavivirus infection, which was thought to be part of the host innate immune response to eliminate invading intracellular pathogens [36,110,111,112]. Because autophagy activation could halt cellular growth and trigger apoptosis, ZIKV-induced autophagy was implicated in the ZIKV-mediated microcephaly [59,111,112]. Activation of autophagy elicits antiviral activities by removing viral proteins through reticulophagy, a selective form of autophagy that leads to ER degradation, or inclusion of viral proteins in autophagosomes destined for lysosomal degradation [113]. The ER-localized reticulophagy receptor FAM134B serves as a host cell restriction factor to ZIKV and other flaviviruses [114]. However, ZIKV-induced autophagy could be a double edged sword, which shows activities of both pro- and anti-ZIKV infection [113]. Activation of cellular autophagy counteracts ZIKV infection by actively removing viral proteins. As part of the host cell’s antiviral responses, type I IFN signaling also limits ZIKV replication by promoting autophagic destruction of the viral NS2B/NS3pro protease in a STAT1-dependent manner [115]. Conversely, ZIKV takes advantage of autophagosome formation, whose presence was associated with enhanced viral replication [64]. ZIKV activates autophagy through the cellular mTOR stress pathway that connects oxidative stress and reactive oxygen species (ROS) production. This virus–host interaction appears to be highly conserved, as in human fetal neural stem cells, ZIKV triggers autophagy through inhibition of the mammalian mTOR pathway via AKT [111]. Similarly, in fission yeast cells, the ZIKV effect on TOR is mediated through a parallel pathway via Tor1 and Tip41, the human equivalents of TSC1 and TIP41 proteins [116,117]. Altogether, ZIKV infection elicits RIG-1/MDA5- and TLR3-mediated innate immune responses leading to releases of type I and type III IFNs to protect cells from viral invasion. ZIKV concurrently triggers cellular activation of the stress TOR signaling pathway that induces autophagy. The balance between pro- and anti-ZIKV activities of autophagy, at least in some cells, determines whether infected cells are protected through viral elimination, or destined to apoptosis as the result of viral propagation in host cells.

## 4. Viral Counteraction to Host Antiviral Responses and ZIKV-Induced Cytopathic Effects

### 4.1. Viral Counteraction to Host Antiviral Responses

To establish successful viral infection, ZIKV has adopted various strategies to counteract host antiviral responses (Table 3). The final infection outcome depends on the balance between the host antiviral responses and the viral counteracting actions. A number of ZIKV-mediated counteracting actions are known. For example, once ZIKV infection is successfully established, it becomes resistant to IFN treatment, suggesting ZIKV might have deployed effective counteractive measures against host innate immune responses [101,125]. Resultant to this finding, no secreted type I and type III IFNs were detectable from ZIKV-infected cells [65]. Indeed, ZIKV impairs the induction of type I IFN by binding to IRF3, a member of the IRF family [49,125,128]. These ZIKV-mediated counteracting effects are achieved through multiple non-structural ZIKV proteins (NS1, NS2A, NS2B, NS4A, NS4B, and NS5). All of these ZIKV proteins suppress, to various degrees, IFN-β production by targeting distinct components of the RIG-I pathway [49]. For instance, the NS1, NS4A, and NS5 proteins specifically inhibit IRF3 and NFkB [125], and the NS1 and NS4B proteins block IRF3 activation [49,115]. Interestingly, an A188V mutated NS1, which was found during the ZIKV epidemic starting in 2012, showed enhanced ability to block IFN-β induction, and facilitated mosquito-mediated virus transmission [49]. This acquired mutation enables NS1 binding to TBK1 and reduces TBK1 phosphorylation. Reversion of this mutation to the pre-epidemic genotype weakens the ability of ZIKV to counteract IFN-β production. Consistent with the idea that ZIKV blocks the IFN-β production through IRF3, IRF3 knockout cells lost this ZIKV effect [48,49].

ZIKV has also developed mechanisms to block the JAK/STAT pathway [65] (Figure 2, middle). For example, it blocks JAK1/Tyk2-mediated STAT1 and STAT2 phosphorylation resulting in ISGF3 transcription and ISGS translation shutdown [65]. On one hand, ZIKV utilizes its PR to inhibit JAK1 kinase [115]. On the other, ZIKV uses NS5 protein through direct binding to promote STAT2 proteasome-mediated degradation [125,126,128].

### 4.2. ZIKV-Induced Cytopathic Effects

Persistent viral replication and propagation inevitably confer adverse CPEs to host cells (Table 4). Like many other viruses, ZIKV encodes a limited number of proteins and, conceivably, has to rely on host cell resources to ensure its successful viral reproduction. Thus, a variety of devious approaches are utilized in order to commandeer host cell resources to create an environment for its own benefit. One common viral strategy is to deter host cell growth, or to subvert the host cell cycle into a specific phase whereby the virus gains optimal benefit by maximizing availability of cellular resources for its transcription, translation and assembly. This indeed is true for ZIKV in that ZIKV infection of hNPCs restricts cell growth and induces cell cycle dysfunction and apoptosis [42,61,63]. Further, these ZIKV-mediated CPEs appear to be associated with clinical neurological manifestations such as microcephaly [42,129]. For instance, ZIKV-induced CPEs correlate with the decrease of neuronal cell-layer volume of the brain organoids reminiscent of processes resulting in microcephaly, supporting that ZIKV-induced microcephaly is likely the result of ZIKV-mediated increase of CPEs [40,43,57,59]. 

Although ZIKV confers various CPEs as described above, the identity of which ZIKV protein(s) is responsible, and the mechanism by which ZIKV mediates those effects, remains elusive. To assist in identifying which ZIKV viral protein(s) is responsible for those observed CPEs, we performed a genome-wide analysis of ZIKV proteins by using fission yeast (*Schizosaccharomyces pombe*) as a surrogate system [9,135]. Fission yeast is particularly useful here because the aforementioned ZIKV-mediated CPEs affect highly conserved cellular activities among all eukaryotes [136,137,138,139]. Each of the fourteen ZIKV viral cDNA encoding a specific protein or a small peptide was cloned into previously described fission yeast gene expression systems [140,141]. All of the ZIKV viral activities were measured simultaneously under the same inducible conditions, thus allowing concurrent functional characterization of each ZIKV protein. Consistent with the idea that ZIKV is a cell membrane-associated virus, and that the ER is the major “viral factory” [36,110,142], nine of the fourteen ZIKV proteins and peptides were found to associate with the ER network, including the nuclear membrane, ER, and Golgi [36,142,143]. Seven ZIKV proteins, including five mature and immature structural proteins (anaC/C, prM/M, and E), and two non-structural proteins (NS2B and NS4A), conferred a number of the same CPEs as reported in the ZIKV-infected mammalian cells infected by ZIKV [9,36,40,42,43]. Specifically, the ZIKV protein-producing yeast cells displayed restricted cellular growth, cellular autophagy, cell hypertrophy, cell cycle dysfunction, and cell death [9]. As described below, some of the same ZIKV protein-mediated CPEs have also been reported in mammalian cells.

### 4.3. The Structural Proteins

Cytopathic effects induced by ZIKV structural proteins are summarized in Table 4. Briefly, the yeast study showed that both the anaC and C proteins localize to the nuclei, triggering cellular oxidative stress leading to cell death [9]. Consistently, C protein is known to localize in the nucleus for other flaviviruses [144,145]. ZIKV C protein is present in human NPC nucleoli, sub-nuclear structures where ribosome biogenesis takes place, and also plays a role in cellular response to stress [130]. The presence of C protein in nucleoli was associated with activation of ribosomal stress and apoptosis [130]. Deleting part of the C protein prevented nucleolar localization, ribosomal stress, and apoptosis [130].

The E protein is a major viral surface protein that is responsible for the viral entry. Thus, it is a crucial viral determinant for initiating the ZIKV–host interaction. Comparison of E protein sequence and structure with that of other flaviviruses suggest ZIKV E protein is unique among flaviviruses, although some portions of it resemble its counterparts in WNV, JEV, and DENV [146,147]. During flaviviral assembly, E interacts with prM to form the prM-E heterodimers that protrude from the viral surface in the non-infectious and immature viral particles [14]. It is also involved in fusing the viral membrane with the host endosome membrane. As with other flaviviruses, the ZIKV E protein is glycosylated at amino acid N154. The E glycosylation appears to be critical for ZIKV infection of mammalian and mosquito cells, because a glycosylation mutant N154Q diminished oral infectivity by *Ae. aegypti* vector and showed reduced viremia and diminished mortality in mouse models [148]. Interestingly, knockout of E glycosylation does not significantly affect neurovirulence in mouse models [148]. While ZIKV encoding non-glycosylated E protein displayed attenuated and defective neuroinvasion when delivered subcutaneously, it replicated well following intracranial inoculation, suggesting possible involvement of E in passing through the blood-brain barrier [149]. Furthermore, ZIKV viral particles lacking the E protein glycan were still able to infect Raji cells expressing the lectin DC-SIGN receptor, indicating the prM glycan of partially mature particles can facilitate the viral entry [150]. The E protein, specifically its extended CD-loop, may confer viral stability, cell cycle-dependent viral replication, and in vivo pathogenesis, as shortening the CD-loop destabilizes the virus, and Δ346 mutation in this loop disrupts thermal stability of the virus [151].

In DENV, the prM protein forms a heterodimer with the E protein and affects viral particle formation and secretion [14]. The resultant non-infectious and immature viral particles are transported through the TGN, where prM is cleaved by a host protease Furin, resulting in mature infectious particles [15,16]. The transition from prM to M via the cleavage of host protease Furin is required for viral infectivity [11,13]. Therefore, both prM and M play important roles in viral pathogenesis. Consistent with the prM/M activities in host cells, in the yeast study, we showed that both prM and M proteins localize in ER [9]. Similarly, prM also localizes in ER in Vero cells [47]. In addition, the prM protein restricts cellular growth, and affects cell cycling leading to cell death in the yeast [9]. At the time of this writing, no description has yet been reported on the effect of individual prM or M protein on those basic cellular functions in mammalian cells. However, mutational analysis shows that the activity of prM protein contributes to fetal microcephaly [152]. Specifically, evolutionary analysis shows that a S139N substitution in the prM protein has persisted in the circulating ZIKV strains since the 2013 outbreak in French Polynesia to the subsequent spread to the Americas. A single serine(S)-to-asparagine(N) substitution (S139N) in the viral polyprotein of a presumably less neurovirulent Cambodian ZIKV_FSS13025_ strain [153], substantially increased ZIKV infectivity in both human and mouse NPCs, and led to more severe microcephaly in the mouse fetus, as well as higher mortality rates in neonatal mice [152]. Results of this study underscore the important contribution of prM to fetal microcephaly. However, the manner in which prM contributes to microcephaly, and the impact of S139N mutation on the prM function, are presently unknown. It is intriguing to note that residue 139 is actually located in the Pr region of the prM protein. Since neither prM nor Pr are present in the mature and infectious viral particles [15,16], it would be interesting to learn the molecular mechanism underlying the effect of the S139N mutation causing increased viral infectivity.

### 4.4. The Non-Structural Proteins

ZIKV PR, which consists of forty residues of the NS2B cofactor and the NS3pro domain of the NS3 [154], has been actively investigated for its PR activities (Table 4) [134,155,156]. In addition to ZIKV PR-mediated proteolysis for its own replication, ZIKV PR also cleaves the ER-localized reticulophagy receptor FAM134B to counteract host cell restriction through a selective form of autophagy known as reticulophagy [114]. Indeed, depletion of FAM134B by RNAi significantly enhanced ZIKV replication [114]. The production of the same PRs by other flaviviruses causes cell death by apoptosis [157,158]. However, whether ZIKV PR causes apoptosis is presently unknown. The yeast study showed that expression of the *NS2B* gene, which encodes the co-factor of the ZIKV PR, does induces cellular autophagy and cell death [9]. It would be of interest to test if fully active ZIKV PR can induce cell death in yeast and mammalian cells.

The NS4A protein, in conjunction with NS4B, activates cellular autophagy through inhibition of the mammalian TOR pathway via AKT [111]. Similarly, NS4A also inhibits the Tor1 pathway in the fission yeast. Furthermore, the yeast study showed that the inhibitory NS4A effect on TOR was mediated through Tor1 and Tip41, which are the human equivalents of TSC1 and TIP41 proteins [116,117].

Expression of NS2A reduces cell proliferation and causes premature differentiation of radial glial cells in the developing mouse brain [131]. In addition, NS2A interacts with adherens junction (AJ) proteins that are present at the epithelial–endothelial cell junctions, resulting in degradation and malformation of the AJ complex [131]. These NS2A-induced growth defect in the embryonic mouse cortex are unique to ZIKV, as the same effects were not seen in DENV. These NS2A effects could pay a role in the pathogenic mechanism underlying ZIKV infection in the developing mammalian brain [131].

NS1 is a highly conserved protein among flaviviruses. It is an essential viral glycoprotein that plays a major role in virus–host interaction as it participates in viral replication, pathogenesis, and immune evasion [159]. As with other flaviviruses, NS1 is expressed at the cell surface and exists in diverse forms. Intracellular NS1 exists as a dimer that is required for viral replication, whereas the secreted NS1 hexamer interacts with host factors and plays a role in immune evasion [159,160]. Freire et al. [161] first revealed adaptation of the NS1 codon to human housekeeping genes in ZIKV Asian lineage, which could facilitate viral replication in humans. Indeed, an alanine(A)-to-valine(V) amino acid substitution at residue 188 (A188V) of the NS1 protein was acquired by the ancient ZIKV strain since the turning of the century in Southeastern Asia. This A188V-carrying ZIKV strain circulated in that region before dissemination to Southern Pacific islands and the Americas [162]. Residue 188 is located within the interface of two NS1 monomers. However, this A188V substitution does not affect NS1 dimerization, instead increasing its secretability [48]. Strikingly, the A188V-carrying ZIKV epidemic strains were much more infectious in mosquitoes (*Ae. aegypti*) than the earlier Cambodia ZIKV_FSS13025_ strain, resulting in increased NS1 antigenemia. Enhancement of NS1 antigenemia in infected hosts promotes ZIKV infectivity and prevalence in mosquitoes, which could have facilitated transmission during the recent ZIKV epidemics [48]. Consistent with this idea, acquisition of the A188V substitution also correlates with enhanced ZIKV evasion of host interferon induction [49]. 

Interestingly, another pathogenic mutation T233A was isolated from the brain tissue of a ZIKV infected fetus with neonatal microcephaly [47]. The ZIKV NS1 T233A mutation, also located at the dimer interface, was not found in any other flaviviruses. This finding could potentially be significant because wildtype T233 organizes a central hydrogen bonding network at the NS1 dimer interface, while the T233A mutation disrupts this network and destabilizes the NS1 dimeric assembly in vitro [47]. However, the pathogenic potential of this mechanism has not yet been tested. Together, these studies on the NS1 protein suggest that ZIKV has acquired specific mutation(s) that increases its ability to evade host immune responses, and favors persistent viral replication, leading to enhanced viral pathogenicity.

## 5. Concluding Remarks

Since the global ZIKV pandemic in 2015, an unprecedented world-wide effort is being made to understand the ZIKV etiology and its associated human diseases. We have learned a great deal about its epidemiology, genetic diversity, viral pathogenicity, and clinical manifestations that are linked to ZIKV-associated human neurological diseases. In this article, we describe molecular interactions of ZIKV with its host cells. In particular, we briefly outline different cell types and receptors utilized by ZIKV for viral entry and infection. We then describe host cellular and immune responses to fight against ZIKV invasion. In response, ZIKV has adopted various counteracting strategies to defeat those host antiviral responses. The overall balance between host antiviral defenses and viral countermeasures determine the outcome of host cells, and the success of viral propagation and survival. Persistent viral replication and propagation inevitably damage human host cells, tissues, and organs, ultimately resulting in fetal microcephaly and a number of other neurologic disorders. Yet, we have only just begun to understand the molecular mechanisms underlying ZIKV interactions with host cells, and how those interactions relate to the observed neurological disorders caused by those newly adopted pathogenic ZIKVs. Much work is still needed to answer some of those same questions as we asked at the beginning, e.g., (1) what specific virological changes have taken place that transformed the ZIKV from a benign virus to a highly pathogenic virus, (2) how could viral mutations, such as those described in this review, alter the viral pathogenicity enabling recently observed neurological disorders, and (3) what specific changes in ZIKV–host interactions ultimately tilt the balance in favor of enhanced CPEs and viral pathogenicity? Ongoing and future research will no doubt continue to strive to provide answers to these questions. We hope this review will serve as a helpful reference to those who study ZIKV–host interactions, and that the information described herein will encourage additional studies focusing on the molecular mechanisms of this virus.

## Figures and Tables

**Table 1:** Zika viral strains that are known to cause microcephaly or microcephaly-like phenotypes.

ZIKV Strain	Model Used	Host/Location/Year	Microcephaly-Like Phenotypes	Reference
Human fetal tissue or organoid models				
MR766	Human brain-specific organoids	Rhesus monkey/Uganda/1947	Increased cell death and reduced proliferation, resulting in decreased neuronal cell-layer volume resembling microcephaly.	[40]
MR766	Human neurospheres and organoids	Rhesus monkey/Uganda/1947	Growth impairment of neurospheres and organoids	[43]
MR766	Human cerebral organoids	Rhesus monkey/Uganda/1947	Reduction of organoid growth and volume reminiscent of microcephaly via induction of TLR3	[57]
FSS 13025	Human brain-specific organoids	Human/Cambodia/2010	Increased cell death and reduced proliferation, resulting in decreased neuronal cell-layer volume resembling microcephaly.	[40]
ZIKV(BR)	Human organoids	Human/Brazil/2015	Reduction of proliferative zones and disrupted cortical layers; induction of apoptosis, autophagy and impaired neurodevelopment	[59]
KU527068	Aborted human fetal brain	Human/Brazil/2016	Microcephaly with calcification in the fetal brain and placenta	[32]
FB_GWUH	Aborted human fetal brain	Human/USA/2016	Fetal brain abnormalities with diffuse cerebral cortical thinning	[39]
Mouse models				
PF/2013/KD507	Mouse	Human/French Polynesia/2013	Fetal demise or intrauterine growth restriction	[33]
ZIKV(BR)	Mouse	Human/Brazil/2015	Intrauterine growth restriction, including signs of microcephaly and vertical transmission	[59]
SZ01	Mouse vertical transmission	Human/Samoa/2016	Infection of radial glia cells of dorsal ventricular zone of the fetuses resulting in reduced cavity of lateral ventricles and decreased cortical surface area	[40]
SZ01	Embryonic mouse brain	Human/Samoa/2016	Cell cycle arrest, apoptosis, and inhibition of NPC differentiation, resulting in cortical thinning and microcephaly	[61]
CAM/2010AndVEN/2016	Neonatal mouse brain	Human/Cambodia/2010 Human/Venezuela/2016	Neonatal ZIKV infection of VEN/2016 leads to more severe microcephaly than CAM/2010. VEN/2016 strain infection leads to stronger immune response, more severe calcification, more neuronal death and abolished oligodendrocyte development, but less activation of microglial cells.	[62]

**Table 2:** Cellular targets and receptor usages.

Primary Cell	Receptor		References
Brain			
Neural progenitor cells (NPCs)	AXL, TLR3		[80,84,85]
Astroglial cells	AXL		[36,81,86,87,88]
Microglial cells	AXL		[81]
Placenta			
Hofbauer cells	AXL, Tyro3, TIM1		[67,68,83]
Trophoblasts	AXL, Tyro3, TIM1, TLR3, TLR8		[67,68,83]
Endothelial cells	AXL, Tyro3, TIM1		[33,83]
Skin			
Dermal fibroblasts	AXL, TIM-1, TYRO3, TLR3, RIG-I, MDA5		[64,89]
Epidermal keratinocytes	AXL, TIM-1, TYRO3, TLR3, RIG-I, MDA5		[64]
Immune cells			
Immature dendritic cells	DC-SIGN		[64,65]
Dendritic cells	DC-SIGN		[66]
CD14^+^ monocytes	Unknown		[90,91,92]
CD14^+^CD16^+^ monocytes	Unknown		[91]
Testis			
Sertoli cell	AXL		[28,93,94]
Spermatozoa	Tyro3		[95,96]
Kidney			
Renal mesangial cell	Unknown		[97]
Glomerular podocytes	Unknown		
Renal Glomerular Endothelial Cell	Unknown		
Retina			
Retinal pericytes	Tyro3, AXL		[1,98]
Retinal microvascular endothelial cells	Tyro3, AXL		
Permissive human cell lines			
Cell line	Origins	Permissiveness	References
SK-N-SH	Brain/Bone marrow	**	[99]
SH-SY5Y	Nerve	**	[100]
SF268	CNS in brain	***	[42,70]
HBMEC	Brain	***	[69,94]
SNB19	CNS in brain	***	[42]
Huh-7	Liver	***	[70]
HFF-1	Skin	***	[64]
A549	Lung	***	[100,101]
HOBIT	Osteoblast-like Cells	***	[102]

Note: **, moderate permissive; ***, highly permissive.

**Table 3:** Cellular antiviral responses and viral counteractions during Zika infection.

Cellular Antiviral Responses to Zika Infection			
Cellular Response	Cellular Protein Involved	Molecular Actions and Consequences	References
Pro-inflammatory CD8+ T-cell immune response	Cytokines: IL-1β, IL-6, MIP1α; chemokines: IP-10, RANTES	T-cell mediated polyfunctional immune responses with releases of antiviral cytokines and chemokines	[65,103,118,119]
CD14+ monocytes and macrophages immune response	CXCL9, CXCL10, CXCL11, CCL5, IL-15	CD14+ monocytes prime NK cell activities during ZIKV infection	[92]
Humoral immune response	IgM, IgG	Production of neutralizing and protective antibodies to ZIKV	[120,121,122]
Cellular innate immune response: TLR3-mediated response	TLR3, IRF3, TBK1, type I IFNs, and IFNβ	An early response that triggers IRF3 and recognizes ZIKV dsRNA in cytoplasm leading to activation of type I IFNs and IFNβ production	[57,64,65]
Cellular innate immune response: RIG-1/MDA5-mediated response	RIG-1, MDA5, IRF-3, NFkB, type I IFNs, and IFNβ	Late responses that recognize ZIKV dsRNA and contribute to activation of type I IFNs and IFNβ production	[64,65]
Type I and type III interferon activation	OAS2, ISG15, MX1	Production of IFNβ as part of the cellular antiviral responses	[64]
**Viral Counteraction**			
Viral response	Viral protein involved	Molecular actions and consequences	References
Counteraction to activation of type 1 IFNs and IFNβ production	NS1, NS2A, NS2B, NS4A, NS4B and NS5	Targeting RIG-1 pathway	[123,124,125]
Inhibition of IFNβ production	NS1, NS4A, NS4B, NS5	NS4A and NS5 inhibit IRF3 and NFkB; NS1 inhibits IRF3 IFNβ production through binding to TBK1	[49]
Inhibition of the JAK/STAT pathway	NS5, PR	NS5 binds to STAT2 for its proteasomal degradation; PR inhibits JAK1 kinase	[115,126,127]
Selective activation of type II IFN signaling	NS5	NS5 promotes the formation of STAT1/STAT1 homodimers and activates type II IFN for viral replication	[123]
Induction of cellular autophagy	prM, M, NS1, NS2A, NS4A	In a yeast study, these ZIKV proteins induced cellular autophagy as indicated by formation of cytoplasmic puncta	[9]
Induction of cellular autophagy	NS4A, NS4B	Inhibit Akt-mediated mTOR pathway through Tor1/TSC1 and Tip41	[9,111]

**Table 4:** ZIKV proteins and associated cytopathic effects.

Protein	Primary Function	Main Phenotypes	References
Structural Proteins			
anaC	Anchored capsid protein	In the fission yeast cells, it restricts cellular growth and affects cell cycling. It also induces cellular oxidative stress leading to cell death.	[9]
C	Capsid protein	In the fission yeast cells, it restricts cellular growth. It also induces cellular oxidative stress leading to cell death; in hNPCs, it induces ribosomal stress and apoptosis.	[9,130]
prM	Precursor membrane protein	In the fission yeast cells, it restricts cellular growth and affects cell cycling. It also induces cellular oxidative stress and autophagy leading to cell death; a single prM mutation contributes to fetal microcephaly	[9,131]
M	Membrane protein	In the fission yeast cells, it restricts cellular growth and affects cell cycling. It also induces cellular oxidative stress and autophagy, leading to cell death.	[9]
Pr	Cleaved product from prM	Unknown	
E	Envelope protein	A putative cytopathic factor based on a yeast study. E protein facilitates viral entry. A single residue in the αB helix of the E protein is critical for Zika virus thermostability, and interaction with the host cell membrane.	[9,132]
Non-structural Proteins			
NS1	Viral replication, pathogenesis and immune evasion	In the fission yeast cells, it induces cellular oxidative stress and autophagy leading to cell death; An essential role in viral replication and immune evasion. It presents on the cell surface and presents as a dimer within cells, and as a hexamer once being secreted. NS1-mediated CPEs in mammalian cells have not yet been established.	[47,48,49,133]
NS2A	Unknown	In the fission yeast cells, it induces cellular oxidative stress and autophagy leading to cell death; ZIKV-encoded NS2A disrupts mammalian cortical neurogenesis by degrading adherens junction (AJ) proteins, leading to reduced proliferation and premature differentiation of radial glial cells and aberrant positioning of newborn neurons.	[131]
NS2B	Protease cofactor	In fission yeast cells, it restricts cellular growth. Forms a protease complex with NS3; a putative cytopathic factor based on a yeast study	[9,134]
NS3	Protease and helicase	NS3-mediated CPEs in mammalian cells have not yet been established.	[131]
NS4A	Viral RNA synthesis and viral morphogenesis	In the fission yeast cells, it restricts cellular growth and affects cell cycling. It also induces cellular oxidative stress and autophagy leading to cell death. It induces autophagy by inhibiting Atk-mediated TOR pathway through Tor1/TSC1 and Tip41 in both yeast and mammalian cells.	[9,111]
2K	A signal peptide	Viral RNA synthesis and viral morphogenesis. 2K-mediated CPEs have not yet been established.	[9,11,12]
NS4B	Viral RNA synthesis and viral morphogenesis	Synergistic to NS4A on inhibiting Akt-mediated TOR pathway	[111]
NS5	Methyltrasferase; RNA-dependent polymerase	NS5-mediated CPEs in mammalian cells have not yet been established.	[128]

## Abbreviations

ADE	Antibody-dependent enhancement
CPE	Cytotoxic effect
CNS	Central nerve system
DC	Dendritic cell
DENV	Dengue virus
dsRNA	Double stranded RNA
ER	Endoplasmic reticulum
HAVcr-1	Hepatitis A virus cellular receptor 1
hEC	Human epithelial cell
hNPC	Human neural progenitor cell
hBMEC	Human brain microvascular endothelial cell
GBS	Guillain–Barré syndrome
IFN	Interferon
iPSC	Induced pluripotent stem cell
IRF3	Interferon regulatory factor 3
ISGS	Interferon stimulated genes
JEV	Japanese Encephalitis Virus
NPCs	Neural progenitor stem cells
PAMP	Pathogen-associated molecular pattern
PR	Protease
PRRs	Pattern recognition receptors
RdRP	RNA-dependent RNA polymerase
RLRs	RIG-I like receptors
sfRNA	Subgenomic flavivirus RNA
TGN	Trans-Golgi network
TLR3	Toll-like receptor 3
TOR	Target of rapamycin
WHO	World health organization
WNV	West Nile Virus
ZIKV	Zika virus
